# Efficacy of the CerveraTS Plate in an Integrated Approach to Juvenile Idiopathic Arthritis: A Long-Term Case Report

**DOI:** 10.1155/crid/1769950

**Published:** 2025-10-30

**Authors:** Valentina Bertolami, Lucia Pozzan, Luca Contardo, Bachar Reda

**Affiliations:** Department of Medical, Surgical and Health Sciences, School of Dentistry, University of Trieste, Trieste, Italy

**Keywords:** Cervera plate, functional appliance, juvenile idiopathic arthritis, TMJ

## Abstract

Juvenile idiopathic arthritis (JIA) is the most common chronic rheumatic disease of unknown etiology affecting children. Temporomandibular joint (TMJ) involvement has been reported in up to 96% of children with JIA. This paper presents a case of JIA with TMJ involvement, emphasizing the clinical features and therapeutic management of the disease. This is a case of a 10-year-old female who presented with pain localized at the left mandible angle during end-feel assessment. During examination, reduced range of motion (ROM) and limited right lateral excursion were observed. Afterwards, a comprehensive multidisciplinary evaluation led to diagnosing the patient with oligoarticular JIA involving the TMJ. Treatment modality included cognitive behavioral therapy, left-sided joint distraction exercises to reduce intra-articular pressure and relax affected masticatory muscles, and a modified CerveraTS plate. Three months of posttreatment follow-up showed pain remission, improved ROM, and enhanced right lateral excursion. Sustained therapeutic outcomes were reported after 1 and 3 years of follow-up visits. The case emphasizes the importance of early diagnosis of JIA with TMJ involvement and tailored management using functional appliances such as the CerveraTS plate which has proved its efficacy in improving mandibular function and pain resolution.

## 1. Introduction

Juvenile idiopathic arthritis (JIA) is the most common chronic rheumatic disease in childhood, with an onset before 16 years of age and persistence for over 6 weeks [1–3]. It is more common in females, with a female-to-male ratio ranging from 3:1 to 6:1 [4, 5]. JIA involving peripheral arthritis, which particularly affects the knees, wrists, and ankles, is the most common form of JIA [1]. According to the International League of Associations of Rheumatology (ILAR), JIA is categorized into seven groups [2], where 40%–96% of patients with JIA develop arthritis of the temporomandibular joint (TMJ), with all subgroups being at risk [6–9].

The exact etiopathogenesis is still unclear, though immunogenic mechanisms triggered by various genetic and environmental factors are well documented. Common etiological factors include stress, trauma, and infections [10–12]. Due to the condition's broad spectrum of clinical presentations, the varying age of onset, and the lack of specific confirmatory tests complicate diagnoses, necessitating a multidisciplinary team approach [2]. This team includes pediatricians, rheumatologists, general dentists, orthodontists, and oral and maxillofacial surgeons.

Treatment modalities may include a combination of approaches such as cognitive behavioral therapy (CBT), physical therapy, nonsteroidal anti-inflammatory drugs (NSAIDs), corticosteroids, biologic treatments, orthodontic interventions, oral appliances, and orthognathic surgery [13–15]. The primary therapeutic goals focus on the early identification of the condition, reduction in disease activity, clinical remission, prevention of further damage, minimizing drug-related side effects, and ultimately ensuring that the patient's quality of life is comparable to that of their healthy peers [16–18].

**Figure 1:**
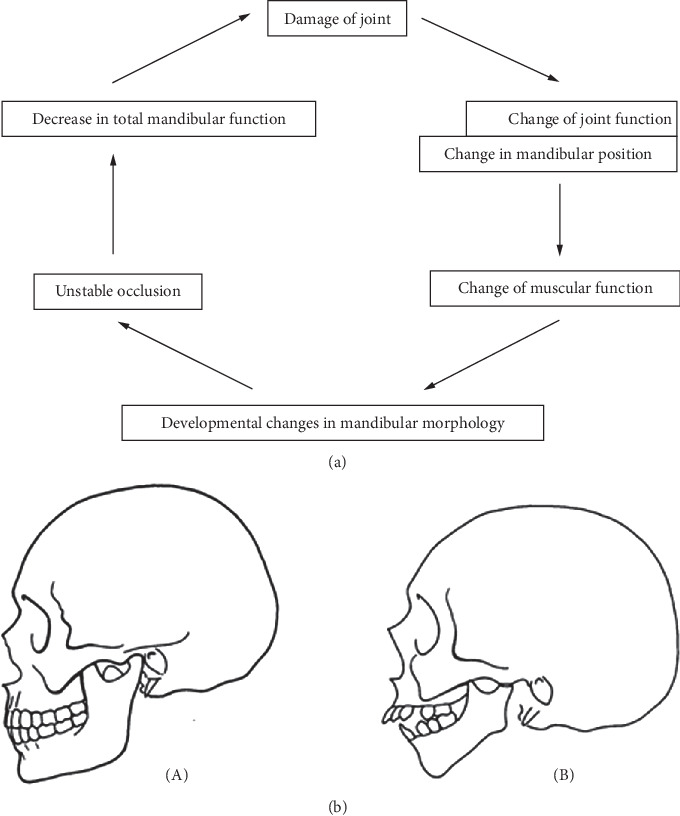
TMJ damage. (a) Flow chart of TMJ damage. (b) Anatomical changes of mandibular bone ((A) pre-TMJ damage and (B) post-TMJ damage).

**Figure 2:**
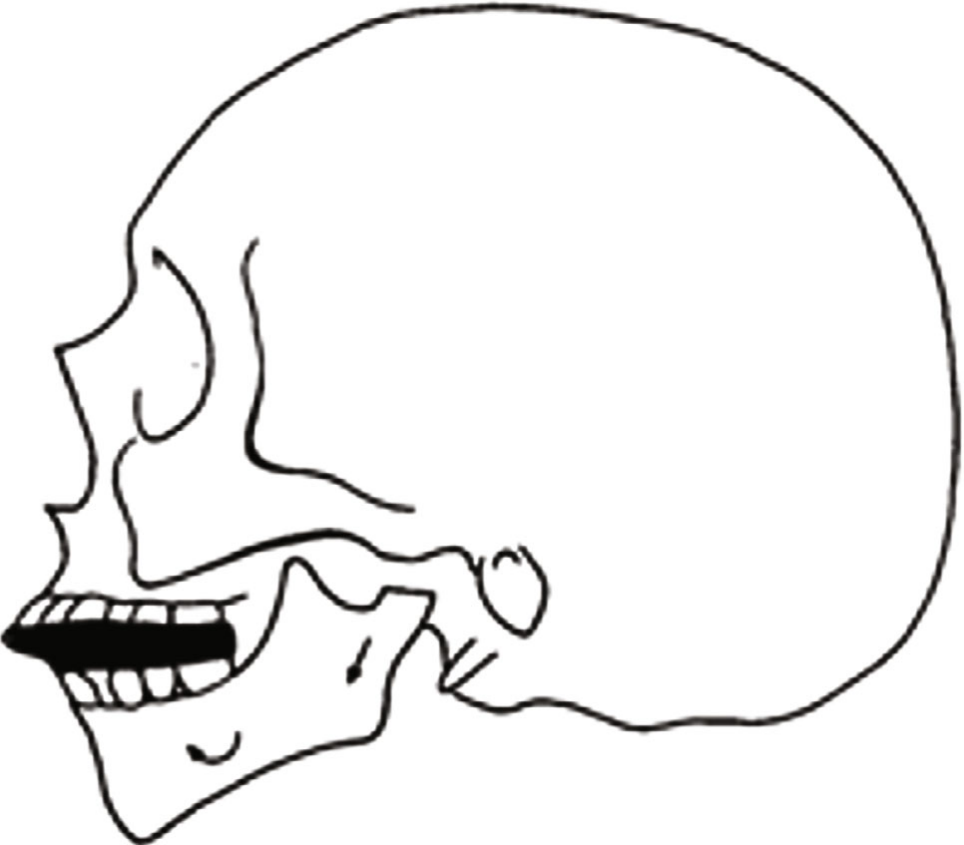
Clinical effects of functional splint.

The treatment of JIA with TMJ involvement lacks sufficient data and guidelines, though many experts recommend incorporating functional devices into the therapeutic plan [9]. Functional splints, for example, have been shown to help prevent further joint damage. Pedersen et al. highlighted how joint damage, including condylar resorption, leads to instability, postrotation of the occlusal plane, and the development of an anterior open bite [19]. These changes can reduce mandibular function and worsen joint damage (Figure 1a,b). Functional splints can interrupt this process by stabilizing the occlusion, promoting condylar distraction, unloading the TMJ, and restoring jaw anterotation (Figure 2). This helps protect the joint, reduce inflammation during the active phase, and stimulate condylar growth during remission.

## 2. Case Presentation

A 10-year-old female patient was referred to the SC (UCO) Clinic of Maxillofacial Surgery and Odontostomatology in Trieste, Italy, due to the onset of pain during mouth opening and observed mandibular deviation during movement.

**Figure 3:**
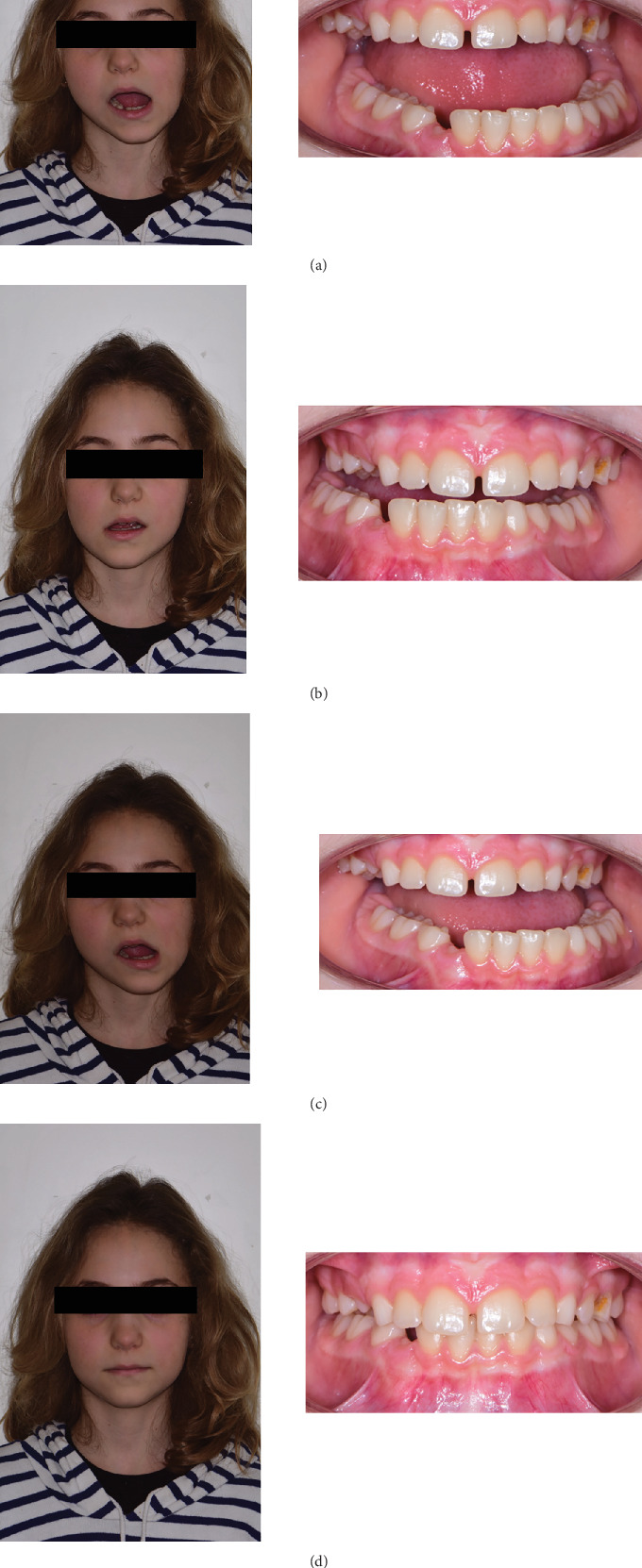
(a) Extra- and intraoral view of mandibular kinetics during the phase of maximum opening of the patient at the first visit. (b) Extra- and intraoral view of mandibular kinetics during the phase of right lateral movement of the patient at the first visit. (c) Extra- and intraoral view of mandibular kinetics during the phase of left lateral movement of the patient at the first visit. (d) Extra- and intraoral view of the patient at the first visit.

**Figure 4:**
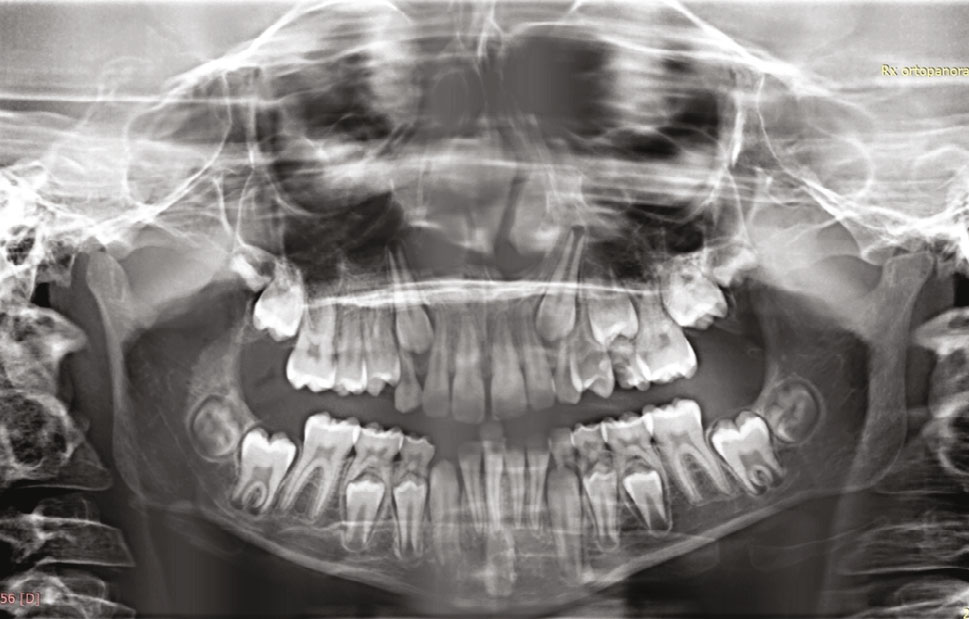
Initial OPG x-ray.

The patient had mixed dentition and coinciding interincisal midlines when the mouth was closed. Deviation toward the left side was evident during mandibular opening, suggesting a unilateral disease. Lateral movements revealed a reduced range of motion (ROM) on the right side, while left-side movements remained normal. Pain was localized at the left mandibular angle during end-feel assessment. Facial asymmetry was visible, characterized by a deficiency on the left mandibular side compared to the contralateral side (Figures 3a, 3b, 3c, and 3d). A comprehensive medical history revealed no systemic diseases, medication use, or trauma to the orofacial complex. An orthopantomogram (OPG) suggested mandibular asymmetry and suspected resorptive changes in the left condyle (Figure 4).

A detailed history of orofacial pain was taken following Axis I and Axis II protocols of the Diagnostic Criteria for Temporomandibular Disorders (DC/TMD) [20]. Psychosocial factors did not appear to influence the patient's clinical symptoms. Additionally, oral behaviors were assessed but were not excessive compared to population norms [21]. At T0, the ROM recorded was 25 mm for passive opening, 31 mm for active opening, 5 mm for right lateral excursion, and 14 mm for left lateral excursion. No joint sounds were detected, while pain upon palpation of the left mandibular angle was present.

Localized myalgia associated with disc displacement without reduction of the left TMJ, potentially accompanied by intermittent locking was the initial diagnosis [22]. To confirm the diagnosis, second-level imaging, including static and dynamic magnetic resonance imaging (MRI), the gold standard for TMJ arthritis evaluation, was ordered [23]. Counseling was provided to the patient and her parents while emphasizing behavioral corrections such as unilateral chewing on the right and avoidance of harmful oral habits. A pediatric rheumatology consultation was also recommended to rule out systemic conditions.

**Figure 5:**
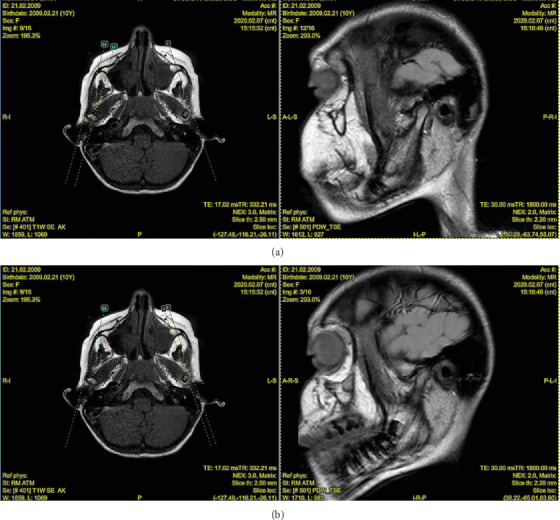
MRI view of the (a) right and (b) left TMJ.

MRI findings and rheumatological assessment led to the exclusion of the initial hypothesis, instead confirming JIA, likely in its oligoarticular form. The MRI revealed significant changes (Figure 5):

• *Right TMJ*: normal condylar morphology, centered position in the glenoid fossa, and normointense fibrocartilaginous tissues (Figure 5a).
• *Left TMJ*: Flattened mandibular condylar head, irregular articular surfaces, and reduced rotational and translational movements during mouth opening (Figure 5b).

Dynamic asymmetry of the mandible was noted in mandibular kinetics. At rest, the chin deviated leftward, with a visibly shorter left mandibular ramus. The mandible deviated to the left without recentering and displayed a limited range (15 mm, compared to a normal range of ~40 mm) during opening [24]. Right lateral movement was also limited (2 mm), while left lateral movement remained within normal limits [24]. Pain was reported at maximum opening, without joint noise.

The treatment plan started with counseling and cognitive–behavioral therapy to mitigate joint overload. Left-sided joint distraction exercises were introduced to reduce intra-articular pressure and relax affected masticatory muscles.

**Figure 6:**
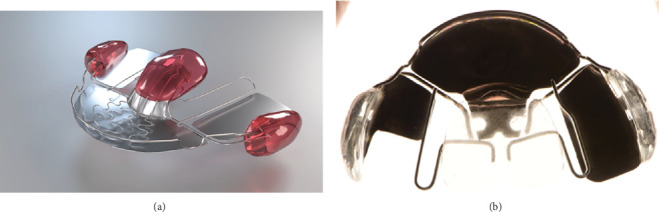
(a) Cervera plate. (b) Modified device (CerveraTS) plate for K.P.

Building on functional appliance concepts, the conventional Cervera plate (Figure 6a) was modified to the *Cervera plate* (CerveraTS) and prescribed. This device included (Figure 6b)

• a palatine resin button with an anterior metal splint
• two wire loops supporting lateral resin shields
• a vestibular arch

The plate was modified to unload the left TMJ by increasing posterior disclusion on the affected side. The appliance aimed to improve occlusal support of the left TMJ while stabilizing mandibular function. The device was worn for 14–16 h daily.

**Figure 7:**
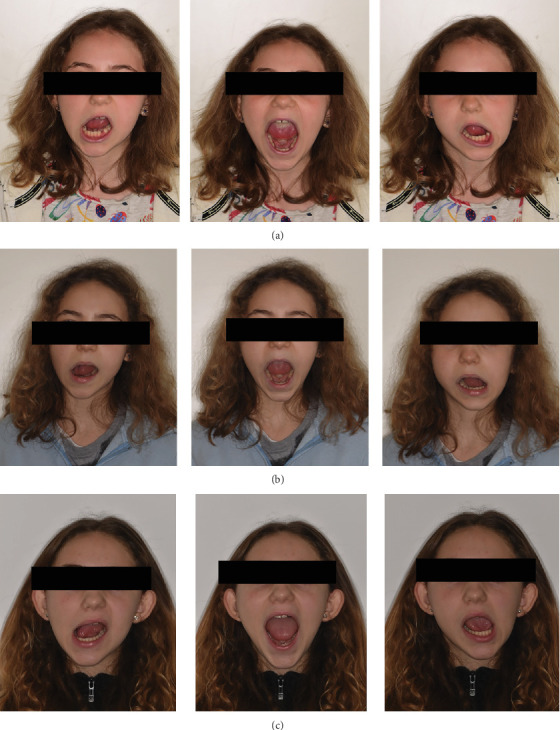
(a) Clinical reevaluation of opening movements and lateral excursions at 3 months after delivery of the CerveraTS plate. (b) Clinical reevaluation of opening movements and lateral excursions at 1 year after delivery of the CerveraTS plate. (c) Clinical reevaluation of opening movements and lateral excursions at 3 years after delivery of the CerveraTS plate.

Upon plate delivery, and by the third month, the patient reported significant improvements in mandibular kinetics and progressive resolution of pain (Table 1). Follow-up photographs demonstrate these improvements at 3 months, 1 year, and 3 years of posttreatment (Figures 7a, 7b, and 7c). Currently, the patient continues to be monitored at the SC (UCO) Clinic. She adheres to prescribed usage of the CerveraTS device and has maintained stability in the absence of symptoms. Fixed orthodontic treatment has been proposed to finalize the case. The patient is also reevaluated periodically by a rheumatologist.

## 3. Discussion

JIA is a chronic condition affecting female children under the age of 16 [1–5]. JIA primarily affects peripheral joints, including knees, wrists, and ankles [1], while 40%–96% of JIA patients develop arthritis of TMJ, with all subtypes being at risk for this complication [6–9]. This case report presents the application of a modified CerveraTS plate in the management of JIA with TMJ involvement, combined CBT, and left-sided joint distraction exercises. The case highlights the efficacy of this approach, demonstrating significant pain resolution and improvement in mandibular function after 3 months of treatment.

A 10-year-old female presented with pain during mouth opening and observed mandibular deviation upon movement. Facial asymmetry, reduced ROM, and limited right lateral excursion were reported during the physical examination, suggesting a unilateral disease. The diagnosis was made through collaboration from a multidisciplinary team, including a general dentist, orthodontist, and pediatric rheumatologist. Orofacial pain was assessed according to the DC/TMD criteria [20], and psychosocial factors were ruled out. MRI and pediatric rheumatology consultation confirmed JIA with TMJ involvement.

Treatment options for JIA with TMJ involvement are limited according to the literature, involving intra-articular corticosteroids, splint therapy, and surgery among the proposed modalities [9]. Based on the available evidence and the patient's diagnosis, the treatment plan comprised CBT and left-sided joint distraction exercises. For splint therapy, a modified CerveraTS plate was chosen, based on the functional appliance concept. According to previous studies, distraction splints have demonstrated efficacy in supporting normal dentofacial growth and limiting the progression of deformities in JIA patients with TMJ involvement [25]. However, bite blocks were typically employed during dental procedures to stabilize occlusion and reduce pain [26]. Functional orthodontic appliances have shown significant improvement in TMJ signs and symptoms over a 24-month period in a patient population [27]. The modified CerveraTS plate was uniquely designed based on the functional appliance concept to unload and reduce intra-articular pressure, which can be achieved through manual therapy, distraction exercises, and occlusal appliances. Additionally, the modified appliance not only served as an occlusal device but also provided the beneficial side effect of posterior molar extrusion, creating a distraction effect with occlusion, even when the device was not worn. This effect was continuous and can be attributed to either the appliance itself or to the enhanced modification, which might have worked synergistically with the distraction exercises.

Follow-up assessments showed significant improvement in pain levels and enhanced mandibular function after 3 months of treatment. These benefits were sustained at both the 1-year and 3-year follow-up visits, suggesting the promising potential of incorporating the CerveraTS plate into the management of JIA with TMJ involvement. The significant effects of this approach were strengthened through the long-term follow-ups as it accounted for the fluctuating course of JIA and reduced the likelihood that improvements were due to spontaneous remission. Long-term interdisciplinary follow-up while integrating rheumatological and orthodontic perspectives is considered essential to optimizing patient outcomes and ensuring continued progress.

## 4. Conclusion

This case highlighted the complexity of diagnosing JIA with TMJ involvement among pediatric patients. Early identification and tailored management using functional appliances such as the CerveraTS plate can significantly improve mandibular function and pain resolution and hence enhance the patients' therapeutic outcomes.

## Figures and Tables

**Table 1:** Timeline changes in mandibular kinetics and symptoms.

	**Upon diagnosis (T0)**	**3-month posttreatment (T1)**	**1-year posttreatment (T2)**	**3-year posttreatment (T3)**
Range of motion (ROM): Passive opening	25 mm	47 mm	47 mm	48 mm
Range of motion (ROM): Active opening	31 mm	47 mm	47 mm	48 mm
Right lateral excursion	5 mm	14 mm	15 mm	15 mm
Left lateral excursion	14 mm	15 mm	15 mm	15 mm
Symptoms	Pain localized at the left mandible angle during end-feel assessment	No	No	No
Joint noise	No	No	No	No

## Data Availability

The data that support the findings of this study are available from the corresponding author upon reasonable request.
